# Investigation of the Protective Effects of Taurine against Alloxan-Induced Diabetic Retinal Changes via Electroretinogram and Retinal Histology with New Zealand White Rabbits

**DOI:** 10.1155/2014/631549

**Published:** 2014-09-14

**Authors:** Samuel Tung-Hsing Chiang, Shang-Min Yeh, Yi-Chen Chen, Shiun-Long Lin, Jung-Kai Tseng

**Affiliations:** ^1^Department of Optometry and Vision Science, Faculty of Medical and Health Science, The University of Auckland, 85 Park Road, Grafton, Auckland 1023, New Zealand; ^2^School of Optometry, Chung Shan Medical University, No. 110, Section 1, Jianguo N. Road, Taichung 40201, Taiwan; ^3^Department of Animal Science and Technology, National Taiwan University, No. 50, Lane 155, Section 3, Keelung Road, Taipei 11054, Taiwan; ^4^Department of Veterinary Medicine, National Chung Hsing University, No. 250, Kuo-Kuang Road, Taichung 40227, Taiwan; ^5^Department of Ophthalmology, Chung Shan Medical University Hospital, No. 110, Section 1, Jianguo N. Road, Taichung 40201, Taiwan

## Abstract

The purpose of this study was to investigate the protective role of orally administered taurine against diabetic retinal changes via electroretinogram (ERG) and retinal histology on rabbits. Rabbits were randomly assigned into groups: Group I (vehicle administration only); Group II (diabetes: induced by 100 mg/kg alloxan injection); Group III (diabetes and fed with 200 mg/kg taurine); and Group IV (diabetes and fed with 400 mg/kg taurine). The body weight and blood glucose levels of the rabbits were monitored weekly. The ERG was measured on weeks 5 and 15. Retinal histology was analyzed in the end of the experiment. Results revealed that a taurine supplement significantly ameliorates the alloxan-induced hyperglycemia and protects the retina from electrophysiological changes. Group II showed a significant (*P* < 0.05) change in the mean scotopic b-wave amplitude when compared to that of Group I, whereas the diabetic rabbits treated with taurine (Group III and IV) were analogous to Group I. Histologically, the amount of Bipolar and Müller cells showed no difference (*P* > 0.05) between all groups and when compared with those of Group I. Our study provides solid evidences that taurine possesses an antidiabetic activity, reduced loss of body weight, and less electrophysiological changes of the diabetic retina.

## 1. Introduction

Diabetes mellitus is one of the most serious medical issues around the world. Untreated diabetes ultimately leads to a variety of secondary complications, such as neuropathy, heart disease, kidney failure, and retinopathy [1]. In the United States, among those adults aged between 20 and 74 years, diabetic retinopathy has been shown to be the leading cause of new cases of blindness [2, 3]. Fong and colleagues [4] described that the prevalence of any signs of retinopathy was as high as 80% at 15 years of having diabetes.

The clinical signs of diabetic retinopathy within the retinal circulation include microaneurysms, haemorrhages, intraretinal microvascular abnormalities, and neovascularization [5, 6]. Microaneurysms are usually the first clinically detectable lesion of diabetic retinopathy; they represent weakening of the capillary walls and may be associated with retinal oedema due to serum leakage from the vessels. Haemorrhages are also an early sign of diabetic damage to blood vessels. They may include “dot and blot” haemorrhages that occur deeper in the retina and shallow flame-shaped haemorrhages that follow the retinal nerve fibre layer. Intraretinal microvascular abnormalities (IRMA) may also be present and caused by poor functioning or nonperfusion of capillaries which prevent normal blood flow. Neovascularization can occur anywhere within the retina as a response to ischaemia and is the hallmark of the advanced and proliferative stage of diabetic retinopathy. The occurrence of neovascularization increases the risk of vision loss in the diabetic patient. In addition to the clinical signs in the retina that can be visualized via ophthalmoscopic view, evidence from previous studies also suggests that choroidal angiopathy may coexist along with retinal vascular damage [7, 8].

Other than the clinical signs mentioned above in detection of diabetic retinopathy, several studies have found that diabetes affects the electrophysiological aspects of vision. Electroretinogram (ERG) is one of the tests that have been well described in the detection of early functional changes in diabetic retinas. In fact, previous studies have demonstrated that ERG abnormalities (i.e., changes in b-wave amplitude) occur before any signs of structural abnormalities can be detected by fundus photography [9], fluorescein angiography [10], and morphological examinations [11].

Taurine (2-aminoethanesulfonic acid) is a conditionally essential amino acid that is present in the retina in a high concentration and is widely distributed in mammalian tissues. The main source of taurine* in vivo* is from a dietary intake of meat or seafood and biosynthesis that is derived from methionine and cysteine metabolism. However, previous study reported that biosynthetic capacity of taurine in humans is very low and absent in cats [12]. Taurine has many biological roles and is involved in several physiological actions, such as the formation of bile acid, osmoregulation, antioxidation, maintaining the structural integrity of the membrane, and modulation of calcium binding and transport [13–15].

In various experimental models, taurine has been shown to protect against alloxan-induced hyperglycemia in type I diabetes [16] and to inhibit cataractogenesis in rabbit lenses exposed to 30 mM galactose [17]. Previous studies have established that taurine is essential for visual development and those deficiencies are associated with retinal degeneration [18]. The physiological role of taurine has been paid attention since reports of cats developing central retinal degeneration when they have been fed to induce a chronic deficiency of taurine, which is similar to the retinitis pigmentosa in humans [19, 20].

Since taurine has been demonstrated to have such excellent bioactivity properties, we hypothesized that taurine administration can protect rabbits from alloxan-induced diabetic retinal changes. The extent of alloxan-induced diabetic retinal changes and protective effects of taurine were measured by electroretinogram (ERG) and histological observations.

## 2. Methods

### 2.1. Animals

Twenty-two male New Zealand White Rabbits (10 weeks old) from Ta Tsung Farm (Changhua City, Taiwan) were used in this study. The animals were quarantined and allowed to acclimatize for one week prior to the experiment phase. The animals were housed one rabbit per cage under standard laboratory conditions with a 12-hour light/dark cycle. The temperature of the animal room was maintained at 25 ± 2°C with a relative humidity of 55 ± 5%. The air-handling units were set to provide approximately 12 fresh air changes per hour. Food and water were available* ad libitum*. The experimental protocols for this study were approved by the Institutional Animal Care and Use Committee (IACUC), Chung Shan Medical University Experimental Animal Center (approval number: 684), and the animals were cared for in accordance with the institutional ethical guidelines. All procedures were performed according to the ARVO Statement for the Use of Animals in Ophthalmic and Vision Research.

### 2.2. Experimental Design

The rabbits were randomly assigned into groups. Group I served as the control (*n* = 6). The experimental groups (Groups II, III, and IV) received an intravenous injection of alloxan [21, 22] in 0.9% sodium chloride at a dosage of 100 mg/kg body weight to induce diabetes. The blood glucose levels were monitored weekly. Those animals with blood glucose levels >200 mg/dL in a consecutive 3-week period were included in the experiment and were distributed into the following groups: Group II diabetes (untreated diabetic rabbits, *n* = 5); Group III (DT200; diabetic rabbits treated with 200 mg/kg taurine in the drinking water, *n* = 5); Group IV (DT400; diabetic rabbits treated with 400 mg/kg taurine in the drinking water, *n* = 6). The food and water intake were checked daily, and body weight was measured weekly.

### 2.3. Blood Glucose Measurement

The blood was collected weekly during 15 weeks of experiment from the marginal ear vein to measure the blood glucose levels (Accu-Chek Active blood glucose meter; Roche Diagnostics Gmbha, Germany).

### 2.4. Electroretinogram (ERG) Analyses

The electroretinogram was measured in both photopic (350 Lux of ambient light) and scotopic (after 5 minutes of dark adaptation) conditions. The instruments used in this study were Eickemeyer ERG, HP Compaq 6230 Notebook, ERG-jet, and ERG-probe by Universo Plastique. The instruments were tested prior to the animals' preparation to ensure that the electrodes were in good order and then the rabbits were prepared in the ambient light with the following steps.

1. One to two drops of tropicamide 1% mydriatic were instilled into rabbits' eyes for at least 30 min prior to ERG.
2. Subcutaneously atropine sulphate was injected at 0.05 mg/kg prior to anesthesia to reduce the salivary and bronchial secretions. Pupils were checked to confirm pupil dilation, and mydriatic drops were repeated at this stage if necessary.
3. Zoletil 50 anesthetics were injected into rabbit's thigh muscles, and then 1 to 2 drops of proparacaine hydrochloride 0.5% local anesthetics were instilled into the rabbits' eyes. Both were done 5 minutes prior to the ERG measurements.
4. Electrodes were placed. Artificial tears were used with contact lens electrode to ensure better contacts of electrode and cornea.
5. Light stimulus was placed 1 cm away from the cornea with standard flash of 2-3 cd/m^2^/s.
6. The electroretinogram was measured with ambient light and after 5 minutes of dark adaptation.
7. Results of ERG including a-wave and b-wave were recorded.

### 2.5. Histological Evaluation

The rabbits were sacrificed at the end of the experiment with 100–150 mg/kg pentobarbital injection and CO_2_; the eyes were removed, weighed, and fixed in Davidson's fixative. The eyes were processed for paraffin embedding following standard microtechniques. Four- to five-micron sections of the eyes tissue were stained with hematoxylin and eosin to estimate the retinal damage and were observed under a microscope (IX71S8F-2, Olympus, Tokyo, Japan).

### 2.6. Statistical Analysis

All results are expressed as mean ± SD. The comparison between any two groups was performed using a one-way analysis of variance (ANOVA), followed by Tukey's multiple comparison tests using the statistical software SPSS (Drmarketing Co., Ltd., New Taipei City, Taiwan). Statistically significant differences between groups were defined as *P* < 0.05.

## 3. Results

### 3.1. Effects of Taurine on Body Weights

**Figure 1:**
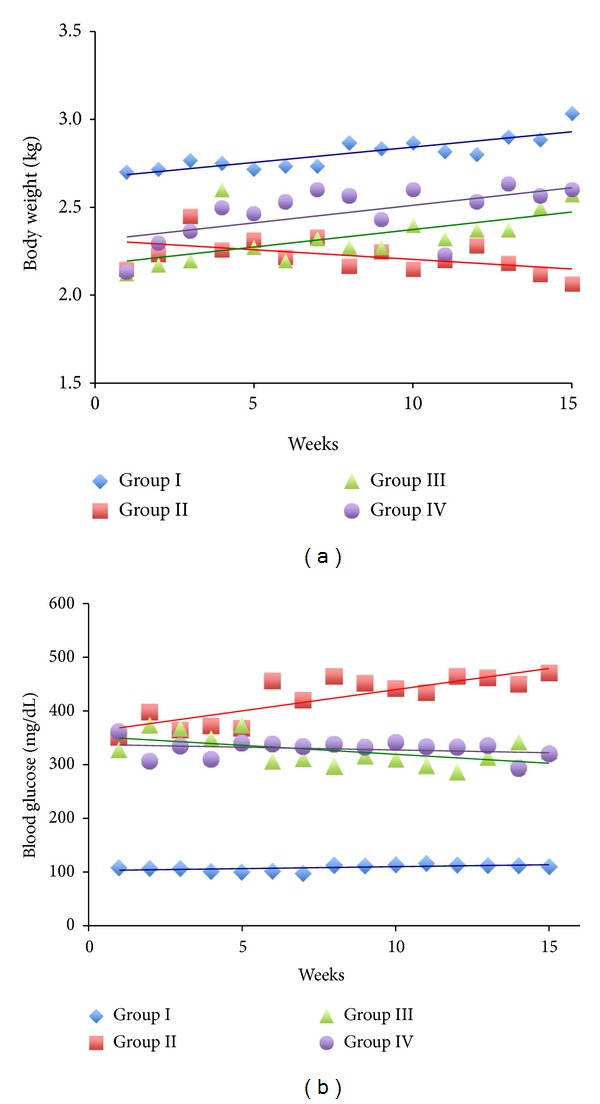
The effect of the taurine on body weight (a) and blood glucose levels (b) in alloxan-induced diabetic rabbits over the 15 weeks of experimentation.

The body weight losses are well diagnosed in alloxan-induced diabetic animal studies. The body weights were recorded weekly over the 15 weeks of study, and the results are shown in Figure 1(a). The trend of the body weight gain revealed an increase in the weights of Group I, Group III, and Group IV over time. Over the 15 weeks of study, the mean increase in body weight for Group I was 0.33 kg, Group III increased by 0.45 kg, and Group IV increased by 0.47 kg. In contrast, Group II lost on average 0.08 kg over the course of the experiment. Although the final body weight of the taurine treated groups was still lower than Group I, they were 24.6% (Group III) and 25.6% (Group IV) heavier than Group II.

### 3.2. Effects of Taurine on Blood Glucose Levels

Blood glucose levels are commonly used as an indicator for alloxan-induced diabetes in experimental animals. They indicate whether or not diabetes was successfully induced. The results of blood glucose levels over the 15 weeks are shown in Figure 1(b). As shown in Figure 1(b), the mean glucose levels of Group I, Group III, and Group IV remained constant over time, whereas Group II's mean blood glucose levels increased over time. This indicated that alloxan is very effective (*P* < 0.0001) in elevating the blood glucose levels in comparison to Group I. This is also as an indication of successfully induced diabetes in rabbits. Our results also demonstrated that taurine treatments alleviate increases of blood glucose levels effectively (*P* < 0.05). On average at 15th week of the study, the glucose levels were lowered by 145 mg/dL (30.9%) in Group III and 151 mg/dL (32.1%) in Group IV when compared to the mean glucose levels of Group II. These findings suggested that the hyperglycemia was induced successfully by alloxan and was effectively ameliorated by taurine treatments.

### 3.3. Electroretinogram

**Figure 2:**
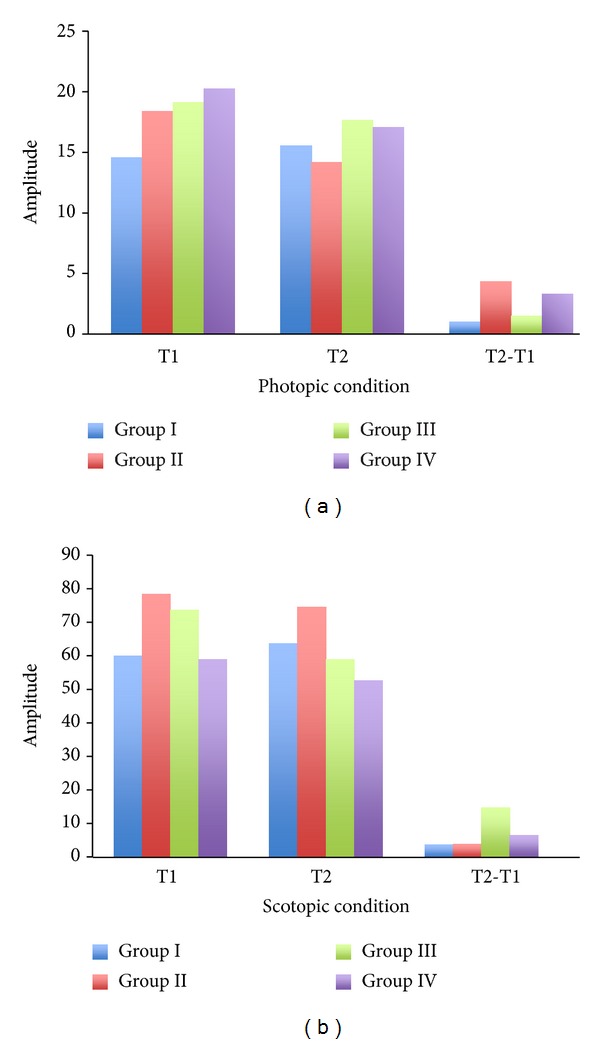
The electroretinogram (ERG) was performed on the control (Group I), diabetes (Group II), and diabetes + taurine (Group III, Group IV) groups on week 5 (T1) and week 15 (T2). The averaged a-wave amplitude under the photopic (a) and scotopic (b) conditions was recorded and analyzed.

**Figure 3:**
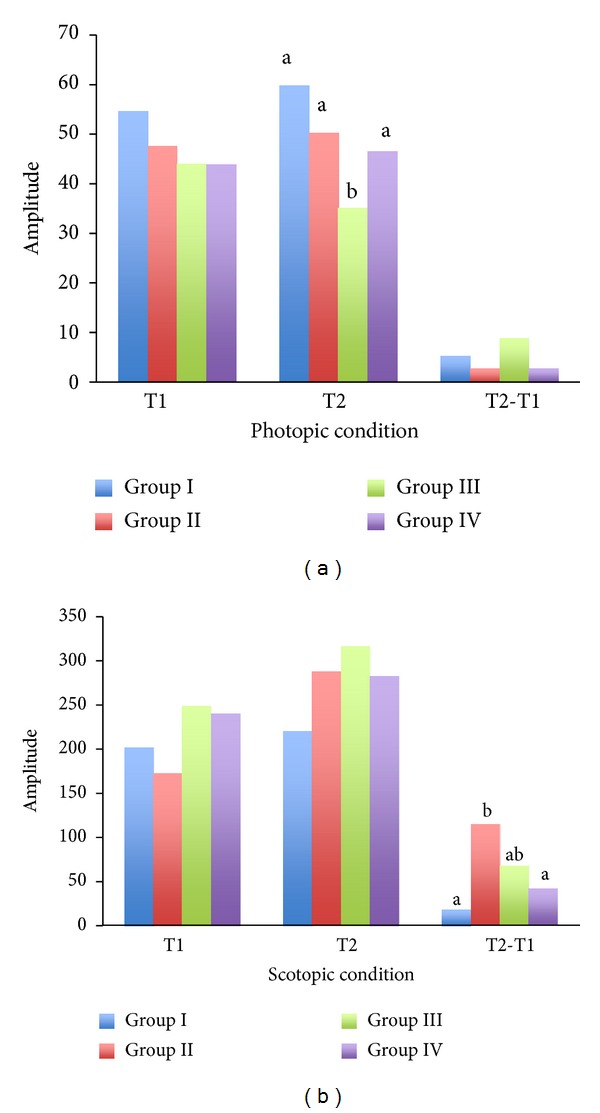
The electroretinogram (ERG) was performed on the control (Group I), diabetes (Group II), and diabetes + taurine (Group III, Group IV) groups on week 5 (T1) and week 15 (T2). The averaged b-wave amplitude under the photopic (a) and scotopic (b) conditions was recorded and analyzed. a, b: bars with different alphabetic letters differ (*P* < 0.05).

The electroretinogram (ERG) was performed for all groups on weeks 5 and 15 to investigate the possible electrophysiological changes of the diabetic retina. The mean amplitude of a-wave and b-wave was analyzed and is shown in Figures 2(a) and 2(b) (a-wave) and Figures 3(a) and 3(b) (b-wave). Over the 15 weeks of study, the mean amplitude changes in both photopic and scotopic a-wave were insignificant (*P* > 0.05). In regard to the b-wave, the mean amplitude under photopic condition was also insignificant (*P* > 0.05). However, the mean amplitude of scotopic b-wave in Group II showed a significantly higher change over time during the experimental period (*P* < 0.05), whilst Group III and Group IV's scotopic changes were not significantly different from animals in the control condition. Group III showed a slightly greater change in scotopic b-wave than Group IV, which may mean that Group IV is more superior in minimizing the electrophysiological changes of the retina over time.

### 3.4. Histological Evaluation

**Figure 4:**
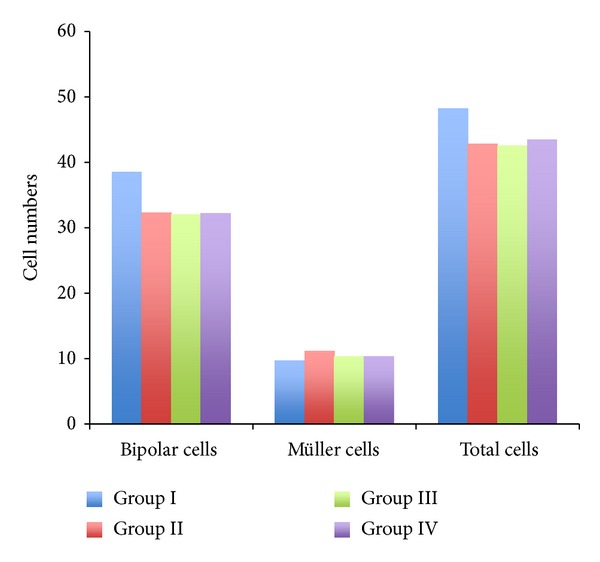
The comparison of Bipolar and Müller cells number among all groups. The cross-section of the retinas was prepared and examined under the microscope for histological evaluation.

The cross-section of the retinas was prepared and examined under the microscope for histological evaluation. The Bipolar and Müller cells were counted and results are shown in Figure 4. Our results revealed that there are not significant differences (*P* > 0.05) in Bipolar and Müller cells' count among all groups when compared with control group. We then further added the Bipolar and Müller cells' count all together to see if there were any clearer trends of cell counts. However, the results still revealed a statistically insignificant difference (*P* > 0.05). These results indicated that there were no obvious cell losses over the 15 weeks of diabetes in rabbits.

## 4. Discussion

Taurine is an organic acid that has many fundamental biological roles in our human body and in animals. Lombardini [23] found that taurine has several functions in the retina, which includes the regulation of Ca^2+^ transport, protection of the photoreceptor, and regulation of signal transduction. A previous study demonstrated that taurine treatments attenuate the induction of retinal VEGF that associates with vascularization in STZ-diabetic rats, suggesting that taurine may normalize the retinal vascular function in diabetes [24]. While elevation of glutamate in the retina is associated with the development of diabetic retinopathy, taurine appeared to be able to regulate Müller cells' glutamate uptake and degradation under diabetic conditions via its antioxidant mechanism [25].

Our study showed that taurine has significant hypoglycemic properties, which is in line with previous work. For example, Gavrovskaya et al. [16] found a decrease in glucose concentration, together with the protection of b-cells of the islets of Langerhans in experimental insulin dependent diabetes mellitus, and other researches have demonstrated the effectiveness of taurine treatments against both insulin dependent and non-insulin dependent diabetes mellitus [26, 27].

In electroretinogram (ERG), the a-wave is a negative, maximal combined response that is believed to reflect the membrane potential in photoreceptors. The b-wave is a positive, maximal combined response that is thought to originate from the Bipolar and Müller cells that postsynapse to the photoreceptors [28]. However, in various animal and human studies, ERG measured under scotopic conditions showed that the large b-wave appeared to be directly generated by the Bipolar cells.

Previous studies investigated the relationship between the ERG changes and diabetic eyes. Holopigian et al. [29] found that the b-wave activity in electroretinogram might indicate retinal changes in early diabetic retinopathy. Coupland [30] and Hardy et al. [31] have demonstrated that the amplitude of the scotopic b-wave reflects the abnormal activity of the Bipolar cells in diabetes even with the absence of visible fundus signs of retinopathy. Kern et al. [32] looked at the streptozotocin-induced diabetes in different species of rats and observed that all strains tended to show diabetes-induced impairment of the dark-adapted (scotopic) b-wave amplitude.

The ERG results from our study revealed that the changes in mean b-wave amplitude of Group II are significantly higher in comparison to Group I, whereas the rabbits with taurine treatments (both Group III and Group IV) reveal no statistical difference against control. Therefore, based on our ERG results, one can assume the following. Firstly, Bipolar cells are probably more susceptible to the alloxan-induced diabetic damages, as the scotopic b-wave appears to be directly generated by the Bipolar cells. Secondly, taurine has protective effects on the diabetic retinas, as it minimizes the retinal electrophysiological changes in the diabetic rabbits. We have also noticed that the rabbits fed daily with 400 mg/kg of taurine had fewer electrophysiological changes over time than the animals assigned to 200 mg/kg of taurine intake. Therefore, the 400 mg/kg taurine supplement may be more superior than the 200 mg/kg of taurine.

In regard to the Bipolar and Müller cells' counts in a retinal histological evaluation, our results revealed no significant difference in Bipolar and Müller cells' counts among all groups (*P* > 0.05). This is probably due to the short experimental period which was unable to observe the retinal histology changes caused by diabetes. This finding confirms previous research findings that the electroretinogram is able to detect abnormalities before any structural changes.

The body weight losses in alloxan-induced diabetes animals were well demonstrated in our study as well. On average, the diabetes group exhibited a 31.7% weight loss when compared with the control group at the end of the 15-week experiment. However, those rabbits treated with 200 mg/kg and 400 mg/kg of taurine daily showed a similar trend of weight gain as the control group over the study period. This also implies that Group III and Group IV end up with much less weight loss compared to those in Group II. Therefore, we can conclude that treatment of taurine protects the alloxan-induced diabetes rabbits from body weight losses.

There are a number of limitations to this study. Firstly, we were unable to determine whether the protective effect of taurine on the retina in diabetic rabbits acted directly on the retina or the effect was indirect and due to hypoglycemic. Secondly, we found no significant differences between the groups for the histological evaluation. This may be because period of 15 weeks is too short to cause the rabbits' retinal tissues change. Future studies could extend the experimental period to further investigate histological differences over time including fluorescein angiography and optical coherence tomography (OCT), particularly for the diabetic group.

In conclusion, our study demonstrated that taurine possesses antidiabetic activities, especially in the hypoglycemic effect, reduces body weight loss, and meanwhile directly or indirectly minimizes the electrophysiological changes in diabetic retina. Future studies are needed to determine whether these results can be replicated in other animal models. If these results hold for human models, taurine could be a cheap and effective way to prevent the damage to retina caused by diabetes.
